# In-Hospital Mortality in Patients with Idiopathic Pulmonary Fibrosis: A US Cohort Study

**DOI:** 10.1007/s00408-019-00270-z

**Published:** 2019-09-20

**Authors:** Michael T. Durheim, Jennifer Judy, Shaun Bender, Dorothy Baumer, Joseph Lucas, Scott B. Robinson, Omar Mohamedaly, Bimal R. Shah, Thomas Leonard, Craig S. Conoscenti, Scott M. Palmer

**Affiliations:** 1 Duke Clinical Research Institute, Durham, NC USA; 2 Duke University Medical Center, PO Box 102355, Durham, NC 27710 USA; 3 Department of Respiratory Medicine, Oslo University Hospital - Rikshospitalet, Oslo, Norway; 4 Premier Inc, Charlotte, NC USA; 5 Boehringer Ingelheim Pharmaceuticals, Inc, Ridgefield, CT USA; 6 Vital Statistics LLC, Chapel Hill, NC USA

**Keywords:** Critical care, Hospitalization, Intensive care, Mechanical ventilation, Interstitial lung disease

## Abstract

**Purpose:**

In patients with idiopathic pulmonary fibrosis (IPF), hospitalizations are associated with high mortality. We sought to determine in-hospital mortality rates and factors associated with in-hospital mortality in patients with IPF.

**Methods:**

Patients with IPF were identified from the Premier Healthcare Database, a representative administrative dataset that includes > 20% of hospital discharges in the US, using an algorithm based on diagnostic codes and billing data. We used logistic regression to analyze associations between patient-, hospital-, and treatment-related characteristics and a composite primary outcome of death during the index visit, lung transplant during the index visit and > 1 day after admission, or death during a readmission within 90 days.

**Results:**

The cohort comprised 6665 patients with IPF hospitalized between October 2011 and October 2014. A total of 963 (14.4%) met the primary outcome. Factors significantly associated with a higher risk of the primary outcome included mechanical ventilation [odds ratio 4.65 (95% CI 3.73, 5.80)], admission to the intensive care unit [1.83 (1.52, 2.21)], treatment with opioids (3.06 [2.57, 3.65]), and a diagnosis of pneumonia [1.44 (1.21, 1.71)]. Factors significantly associated with a lower risk included concurrent chronic obstructive pulmonary disease [0.65 (0.55, 0.77)] and female sex [0.67 (0.57, 0.79)].

**Conclusions:**

Patients with IPF, particularly those receiving mechanical ventilation or intensive care, are at substantial risk of death or lung transplant during hospitalization or death during a readmission within 90 days.

**Electronic supplementary material:**

The online version of this article (10.1007/s00408-019-00270-z) contains supplementary material, which is available to authorized users.

## Introduction

Idiopathic pulmonary fibrosis (IPF) is a progressive fibrosing interstitial lung disease (ILD) characterized by loss of lung function, which occurs mainly in adults over the age of 60 years [1]. IPF has a poor prognosis, with a median survival from the time of diagnosis of approximately 4 years [2]. Hospital admissions are frequent among patients with IPF and are associated with high mortality [3–11], particularly in the case of acute exacerbations of the disease [12].

Diagnostic code-based algorithms have been applied to large administrative databases to conduct population-based studies on the incidence, prevalence and survival of patients with IPF, as well as the economic burden of this disease [2, 4, 8, 13–17]. The use of refined algorithms has enabled identification of patients with IPF with a positive predictive value (PPV) of over 80% [15]. Large administrative databases have also been used to characterize in-hospital mortality in US patients with IPF [4, 8], but previous studies have been restricted by age, a single-payer focus and the limited data collected on patient characteristics and in-hospital treatments.

In this study, we leveraged previously validated algorithms, and addressed limits to their generalizability, by using information from diagnostic codes and billing data to identify patients with IPF in the Premier Healthcare Database (PHD). The PHD is a nationally representative administrative dataset that includes over 20% of hospital discharges in the US, independent of payer, and provides patient-level data on hospital stays, including detailed information on hospital and patient characteristics, diagnostic testing, and treatments. We determined in-hospital mortality rates and the patient-, hospital-, and treatment-specific factors that were associated with in-hospital mortality, urgent lung transplant, or death during a readmission within 90 days of the index visit. In addition, we estimated the median length of hospital stay and identified factors associated with longer hospital stay or readmission.

## Methods

### Study Design

This was a retrospective cohort study of patients with a diagnosis of IPF who were hospitalized at 641 hospitals in the US. Patients were identified using an algorithm that included both diagnosis codes and billing data for chest computed tomography (CT) and/or lung biopsy. Validation of the coding algorithm was performed in a subset of patients through manual review of individual charts (see online supplementary material).

### Patients

Patients with IPF aged ≥ 50 years with a hospital discharge date between 1 October 2011 and 14 October 2014 were eligible for inclusion in the study. Following an approach used in previous studies [2, 15, 17], the diagnosis of IPF was based on a primary or secondary International Classification of Diseases Ninth Revision (ICD-9) diagnosis code of 516.3 or 516.31 and a billing code for chest CT and/or surgical lung biopsy ≤ 3 years prior to the index hospitalization. The period studied was selected based on the introduction of the ICD-9 code of 516.31 for IPF (1 October 2011) and the FDA approval of nintedanib and pirfenidone as treatments for IPF (14 October 2014). Exclusion criteria were any ICD-9 codes for an alternative cause of ILD during the index hospitalization and an ICD-9 procedure code for lung transplant surgery ≤ 1 day after admission. Patients who had lung transplant surgery within a day of admission were excluded, as death following transplant surgery is driven by different factors than death prior to transplant. Patients undergoing transplant > 1 day after admission were included in the analysis, as they were admitted for a reason other than a planned transplant, and transplant surgery that occurs > 1 day after admission likely reflects an urgent, rather than elective, procedure.

### Outcomes

The primary outcome was a composite of in-hospital mortality: death during the index visit, lung transplant during the index visit but > 1 day after admission, or death during a readmission within 90 days of the index visit. Secondary outcomes were length of stay in hospital and readmission to hospital within 90 days of the index visit due to any cause.

### Patient-, Hospital-, and Treatment-Related Characteristics

Patient characteristics analyzed included sex, age, race, and primary payer. Hospital region (South, Midwest, West, North-East of the US), teaching status (academic, non-academic), population served (urban, rural), and attending physician specialty (internal medicine/hospitalist, family medicine, pulmonary medicine, critical care/intensivist, other) were categorized. ICD-9 was used for identification of procedures or concurrent diagnoses in addition to billing descriptions (see online supplementary table). Mechanical ventilation was primarily identified based on invasive mechanical ventilation, but 36 records where it was unknown whether the ventilation was invasive or non-invasive were included. Surgical lung biopsy was identified based on Current Procedural Terminology (CPT) codes and billing descriptions. Chest CT was identified based on CPT codes. Intensive care unit (ICU) use was based on room and board billing. Antibiotics (intravenous [IV], oral), steroids (IV, oral), immunosuppressants (IV, oral), oral anticoagulants, and IV heparin were considered treatments of primary interest; data on use of these treatments were derived from billing descriptions.

### Statistical Analyses

Logistic regression was used to determine associations between patient-, hospital-, and treatment-related characteristics and the primary outcome, as well as between these factors and readmission within 90 days of the index visit. Length of stay was modeled using negative binomial regression and estimates based on patient-, hospital-, and treatment-related factors were calculated. Models were built using bidirectional stepwise variable selection, and an alpha value of 0.01 was considered statistically significant for the purpose of covariate inclusion in the modeling process. We conducted the analyses using a patient-level perspective, in which every patient was counted once, using their first visit during the study period as the index visit.

## Results

### Patients

**Fig. 1:**
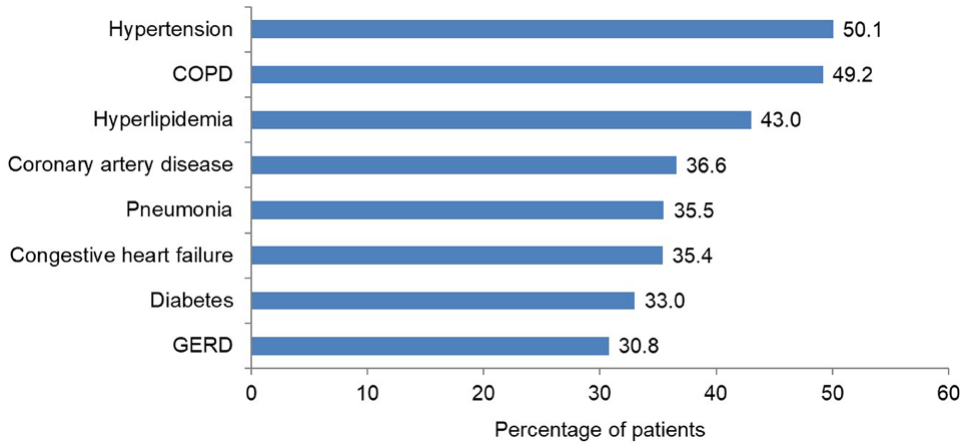
Concurrent diagnoses among patients with idiopathic pulmonary fibrosis identified from a representative US database. Concurrent diagnoses reported in > 30% of patients are shown. *COPD* chronic obstructive pulmonary disease, *GERD* gastroesophageal reflux disease

The cohort consisted of 6665 hospitalized patients with IPF (Table 1). Median age was 75 years and 54.1% of patients were male. Comorbidities and medication use, particularly those related to respiratory and cardiovascular disease, were common (Fig. 1; Table 2). Notably, almost 79% of patients received antibiotics, and the majority received parenteral heparin, and/or oral or parenteral corticosteroids. Over a quarter (28%) of patients received care in the ICU, with mechanical ventilation performed in 10% of patients. Almost 60% of patients received a chest HRCT, while 6.6% and 4.1% received bronchoscopy or lung biopsy, respectively.

**Table 1:** Characteristics of 6665 hospitalized patients with idiopathic pulmonary fibrosis identified from a representative US database

Age, years, median (interquartile range)	75 (67–82)
Male, *n* (%)	3606 (54.1)
Race, *n* (%)	
White	5343 (80.2)
Black	453 (6.8)
Other	869 (13.0)
Primary payer, *n* (%)	
Medicare	5370 (80.6)
Managed care	583 (8.7)
Medicaid	312 (4.7)
Commercial	165 (2.5)
Other	235 (3.5)
Hospital location—US region, *n* (%)	
South	3198 (48.0)
Midwest	1562 (23.4)
West	1061 (15.9)
Northeast	844 (12.7)
Attending physician specialty, *n* (%)	
Internal medicine/hospitalist	4428 (66.4)
Family medicine	584 (8.8)
Pulmonary medicine	453 (6.8)
Critical care/intensivist	126 (1.9)
Other	1074 (16.1)
Admitted to teaching hospital, *n* (%)	2735 (41.0)
Population served by hospital, *n* (%)	
Urban	5850 (87.8)
Rural	815 (12.2)

**Table 2:** Interventions among 6665 hospitalized patients with idiopathic pulmonary fibrosis identified from a representative US database

Diagnostic tests	
Chest HRCT	3946 (59.2)
Echocardiogram	749 (11.2)
Bronchoscopy	440 (6.6)
Lung biopsy	276 (4.1)
Medication use^a^	
Antibiotic	5259 (78.9)
Parenteral heparin	4549 (68.3)
Proton pump inhibitor	4172 (62.6)
Diuretic	3909 (58.6)
Oral or parenteral corticosteroid	3850 (57.8)
Opiate	3235 (48.5)
Statin	3039 (45.6)
Aspirin	2915 (43.7)
Oral anticoagulant	1142 (17.1)
Mechanical ventilation	664 (10.0)
Admission to ICU	1869 (28.0)
Surgery	208 (3.1)

^a^Medications utilized in > 5% of patients are shown. HRCT, high-resolution computed tomography; ICU, intensive care unit

### In-Hospital Mortality

**Fig. 2:**
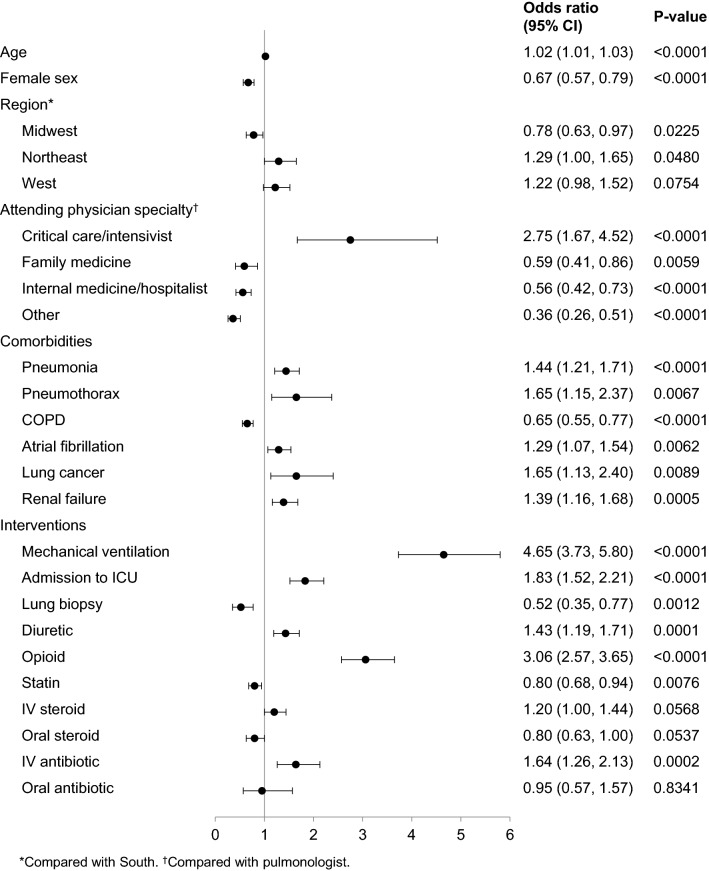
Associations between patient-, hospital- and treatment-related factors and in-hospital mortality or lung transplantation in patients with idiopathic pulmonary fibrosis. *COPD* chronic obstructive pulmonary disease, *ICU* intensive care unit, *IV* intravenous

A total of 963 patients (14.4%) met the primary outcome: 684 patients (10.3%) died during the index visit, 267 patients (4.0%) died during a readmission within 90 days, and 12 patients (0.2%) underwent lung transplantation during the index visit but > 1 day after admission. Factors significantly associated with a higher risk of the primary outcome included mechanical ventilation [odds ratio (OR) 4.65 (95% CI 3.73, 5.80)], admission to the ICU [OR 1.83 (95% CI 1.52, 2.21)], attendance by a critical care physician [OR 2.75 (95% CI 1.67, 4.52)], treatment with opioids [OR 3.06 (95% CI 2.57, 3.65)], and concurrent pneumonia [OR 1.44 (95% CI 1.21, 1.71)] (Fig. 2). The primary outcome was met by 578 (30.9%) of the 1869 patients admitted to ICU, 77 (61.1%) of the 126 patients attended by a critical care physician, 366 (55.1%) of the 664 patients who received mechanical ventilation, 733 (22.7%) of the 3235 patients who received opioids and 485 (20.5%) of the 2368 patients who had pneumonia.

Factors significantly associated with a lower risk of the primary outcome included attendance by an internal medicine [OR 0.56 (95% CI 0.42, 0.73)] or family medicine physician [OR 0.59 (95% CI 0.41, 0.86)], concurrent COPD [OR 0.65 (95% CI 0.55, 0.77)], and female sex [OR 0.67 (95% CI 0.57, 0.79)] (Fig. 2).

### Length of Stay

Median (interquartile range) length of stay in hospital was 5 (3–9) days: 8 (4–14) days for patients who died in hospital and 5 (3–8) days for patients who did not. Patients diagnosed with pneumonia were estimated to have a 15% longer hospital stay than those who were not, and patients diagnosed with pneumothorax were estimated to have a 49% longer hospital stay than those who were not (Table 3). Patients admitted to the ICU were estimated to have a 32% longer hospital stay than those who were not, while patients who received mechanical ventilation were estimated to have a 22% longer stay than those who did not. Patients undergoing bronchoscopy and lung biopsy were estimated to have 32% and 23% longer hospital stays, respectively, than those who did not.

**Table 3:** Estimated lengths of stay in hospital among patients with idiopathic pulmonary fibrosis based on patient-, hospital-, and treatment-related factors

Parameter	Coefficient estimate (SE)	Exponential estimate^a^	*p* value
Insurance (vs medicare)			
Medicaid	0.09 (0.04)	1.09	0.0126
Managed care	− 0.05 (0.03)	0.96	0.0955
Commercial	− 0.06 (0.05)	0.94	0.2095
Other	− 0.07 (0.04)	0.93	0.0839
Region (vs South)			
Northeast	0.03 (0.03)	1.03	0.3201
West	− 0.08 (0.02)	0.93	0.0005
Midwest	− 0.12 (0.02)	0.89	< 0.0001
Rural hospital (vs urban)	− 0.09 (0.03)	0.91	0.0001
Teaching hospital (vs non-teaching)	0.08 (0.02)	1.08	< 0.0001
Attending physician specialty (vs pulmonologist)			
Family medicine	0.06 (0.04)	1.06	0.1143
Other	0.05 (0.04)	1.05	0.1350
Internal medicine/hospitalist	− 0.02 (0.03)	0.98	0.5595
Critical care/intensivist	− 0.29 (0.06)	0.75	< 0.0001
Concurrent diagnoses			
Pneumothorax	0.40 (0.04)	1.49	< 0.0001
Malnutrition	0.32 (0.03)	1.37	< 0.0001
Diverticulosis	0.17 (0.04)	1.19	< 0.0001
Renal failure	0.17 (0.02)	1.18	< 0.0001
Pneumonia	0.14 (0.02)	1.15	< 0.0001
Cerebrovascular disease	0.14 (0.03)	1.15	< 0.0001
Acute heart failure	0.07 (0.02)	1.07	0.0029
Interventions			
Mycophenolate	0.34 (0.06)	1.40	< 0.0001
Admission to ICU	0.28 (0.02)	1.32	< 0.0001
Bronchoscopy	0.28 (0.03)	1.32	< 0.0001
Diuretic	0.25 (0.02)	1.29	< 0.0001
Intravenous antibiotic	0.25 (0.02)	1.28	< 0.0001
Lung biopsy	0.20 (0.04)	1.23	< 0.0001
Mechanical ventilation	0.20 (0.03)	1.22	< 0.0001
Oral antibiotic	0.20 (0.04)	1.22	< 0.0001
Phosphodiesterase type 5 inhibitor	0.19 (0.05)	1.21	< 0.0001
Opioid	0.18 (0.02)	1.20	< 0.0001
Surgery	0.18 (0.04)	1.20	< 0.0001
Anticoagulant	0.14 (0.02)	1.15	< 0.0001
Chest HRCT	0.14 (0.02)	1.15	< 0.0001
Intravenous steroid	0.14 (0.02)	1.15	< 0.0001
Oral steroid	0.11 (0.02)	1.12	< 0.0001
Proton pump inhibitor	0.11 (0.02)	1.11	< 0.0001
Heparin	0.10 (0.02)	1.10	< 0.0001
Echocardiogram	0.09 (0.02)	1.09	0.0004
Prior admission (within past 365 days)	0.08 (0.02)	1.08	< 0.0001

*HRCT* high-resolution computed tomography, *ICU* intensive care unit

^a^Estimated length of stay relative to the reference group

### Readmission

**Fig. 3:**
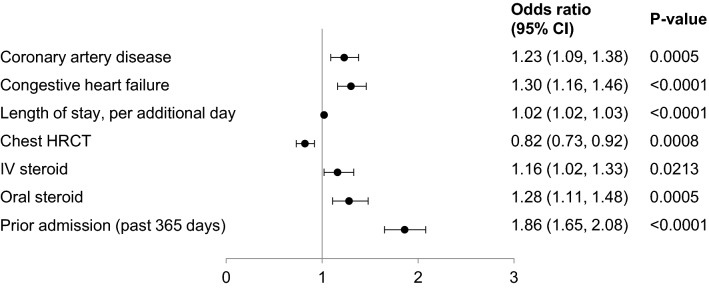
Associations between patient-, hospital- and treatment-related factors and 90-day readmission in patients with idiopathic pulmonary fibrosis. *HRCT* high-resolution computed tomography, *IV* intravenous

A total of 1990 patients (29.9%) were readmitted to hospital within 90 days of the index visit, of whom 267 patients (4.0% of the overall population; 13.4% of those readmitted) died during the readmission visit. Factors significantly associated with a higher risk of 90-day readmission included admission within 365 days prior to the index visit for any reason [OR 1.86 (95% CI 1.65, 2.08)], a longer length of stay in the index visit [OR 1.02 (95% CI 1.02, 1.03)], concurrent congestive heart failure [OR 1.30 (95% CI 1.16, 1.46]), concurrent coronary artery disease [OR 1.23 (95% CI 1.09, 1.38)], use of oral steroids [OR 1.28 (95% CI 1.11, 1.48), and use of IV steroids [OR 1.16 (1.02, 1.33)] (Fig. 3).

## Discussion

In this analysis of 6665 patients with IPF hospitalized across 641 US hospitals, approximately 14% of patients died during the index admission, underwent urgent lung transplant, or died during a readmission within 90 days. As the database used was not limited by patient age, payer, or hospital type, this estimate of in-hospital mortality is broadly representative of US patients with IPF. Similar rates of in-hospital mortality in patients with IPF have been reported in the US Nationwide Inpatient Sample (14%) [8, 18], US Healthcare Cost and Utilization Project Nationwide Readmissions Database (11%) [19], the Spanish National Hospital Discharge Database (14%) [20], and the French hospital discharge database (13%) [7]. A higher rate (23%) was reported in patients with IPF who were hospitalized between April 2010 and March 2013 in the nationwide Japanese database, likely due in part to the exclusion of some elective hospitalizations [9].

Mechanical ventilation was the most striking factor associated with the composite mortality outcome, with an almost five-fold increased odds for death. Consistent with data from the US Nationwide Inpatient Sample [21], more than half of the patients who underwent mechanical ventilation died during admission/readmission or underwent lung transplant. Admission to the ICU or attendance by a critical care physician (which usually occurs in an ICU) was also associated with a significantly increased risk of mortality. Comorbidities associated with an increased risk of mortality included pneumonia, pneumothorax, atrial fibrillation, lung cancer, and renal failure. Identifying prognostic factors, and quantifying the strength of their association with mortality, may assist patients, families, and clinicians in making decisions about their care, particularly regarding the potential benefits and risks of ICU care and mechanical ventilation.

Among treatments, opioids were most strongly associated with mortality, most likely reflecting the palliation of end-stage disease. Other treatments associated with increased mortality included diuretics and IV antibiotics, consistent with the associations between renal failure and pneumonia and mortality. Oral or IV steroids were not associated with improved mortality, while statin use was associated with a slightly reduced risk.

Female patients had a lower risk of in-hospital mortality. The explanation for this finding is unknown. It may be that a greater proportion of female than male patients were assigned a diagnosis code for IPF during their hospitalization when in fact they had connective tissue disease-associated ILD, which is associated with lower mortality than IPF [22, 23]. However, the validated approach used to identify IPF, and the lack of a difference in the PPV of the algorithm between males and females, make this a less likely explanation. Several retrospective cohort studies have shown that mortality is higher in male patients with IPF [10, 24, 25]. A study that used a regression model to identify predictors of mortality in patients with ILD admitted to an ICU for respiratory failure found higher mortality in males [23]. In a mouse model of bleomycin-induced lung fibrosis, males develop increased inflammatory cytokines, collagen deposition, neutrophilic alveolitis, and have higher mortality than females, suggesting that there may be differences between genders in processes that drive progression of fibrosis [26].

Interestingly, in our study, patients with concurrent COPD had a lower risk of in-hospital mortality. This might have been because in some of these patients, it was worsening of COPD, rather than IPF, that led to the hospitalization, and worsening of COPD is associated with lower mortality. Also of interest were some factors that were not associated with mortality in our study, for example, insurance provider, region, and academic status of the hospital.

The median length of hospital stay was 5 days. This is shorter than the lengths of stay reported in previous real-world studies and clinical trials, which have ranged from 7 to 10.2 days [3, 7, 8, 20, 27], likely reflecting differing patient populations and healthcare systems. Length of stay was 32% longer in patients receiving intensive care. Consistent with previous observations [21], LOS was 22% longer among patients receiving mechanical ventilation. These interventions come at considerable economic cost, with a previous US study estimating an increase of over $35,000 per stay associated with invasive mechanical ventilation and over $5000 per stay associated with non-invasive mechanical ventilation [21]. Patients who underwent bronchoscopy or surgical lung biopsy also had longer hospital stays. The reasons for this were not investigated in our study, but may be related to complications such as pneumothorax.

Almost a third of the hospitalized patients with IPF in our analysis were readmitted to a hospital within 90 days. The strongest predictor of readmission was admission within the previous year. These data suggest that there is a subset of patients with IPF who experience frequent hospital admissions; further studies to characterize these patients would be valuable.

Our analyses have several limitations. Our study was limited to the US, which has a different healthcare system to other countries. It is possible that we over- or under-estimated the IPF cohort in our database based on our coding approach; however, the PPV of our algorithm was consistent with previous studies [15]. Using diagnoses of IPF, as well as concurrent conditions, based on administrative data has inherent limitations. For example, it is not possible to determine the extent to which diagnoses of pneumonia reflect concurrent lung infections as opposed to acute exacerbations of IPF. Some factors associated with mortality in patients with IPF (*e.g*. lung function, use of supplementary oxygen) could not be investigated in our analyses. Assessment of readmissions was based only on readmissions to the same hospital, resulting in underestimation of readmission rates and probably also mortality [28]. Deaths outside of hospital were not captured.

Our study covered a period prior to the approval of pirfenidone and nintedanib as treatments for IPF. The use of antifibrotic therapies reduces the risk of acute worsenings of IPF [29–31] and there is increasing evidence that they improve survival [32–37]. Our study provides a basis for future studies looking at the effects of antifibrotic therapies on the outcomes of hospitalizations in a contemporary IPF cohort.

In conclusion, our findings, based on a representative national cohort of hospitalized patients in the US, provide clinically relevant insights into the factors associated with in-hospital mortality in patients with IPF. Patients with IPF, particularly those receiving mechanical ventilation or intensive care, or diagnosed with pneumonia, are at substantial risk of death during hospitalization, lung transplant during admission, or death during a readmission within 90 days. Female patients and those with comorbid COPD appear to be at lower risk of in-hospital mortality. Patients with IPF spend a median of 5 days in hospital, with ICU admission, mechanical ventilation, bronchoscopy, and surgical lung biopsy associated with a longer duration of stay.

## Electronic supplementary material

Below is the link to the electronic supplementary material.

Supplementary file1 (DOCX 87 kb)
