# Supplementary material for: In-Hospital Mortality in Patients with Idiopathic Pulmonary Fibrosis: A US Cohort Study
Source: Lung. 2019 Sep 20;197(6):699–707. doi: 10.1007/s00408-019-00270-z (PMC6861436; doi:10.1007/s00408-019-00270-z)
Supplement: Supplementary file 1 — Supplementary file1 (DOCX 87 kb) [file 408_2019_270_MOESM1_ESM.docx]

**In-hospital mortality in patients with idiopathic pulmonary fibrosis: a US cohort study**

Michael T Durheim, MD,^1,2,3^ Jennifer Judy, PhD,^4^ Shaun Bender, PhD,^5^ Dorothy Baumer, MSc,^4^ Joseph Lucas, PhD,^6^ Scott B Robinson, PhD,^4^ Omar Mohamedaly, MD,^1^ Bimal R Shah, MD,^2^ Thomas Leonard, PhD,^5^ Craig S Conoscenti, MD,^5^ Scott M Palmer, MD^1,2^

^1^Duke Clinical Research Institute, Durham, North Carolina, USA; ^2^Duke University Medical Center, Durham, North Carolina, USA; ^3^Department of Respiratory Medicine, Oslo University Hospital - Rikshospitalet, Oslo, Norway; ^4^Premier Inc., Charlotte, North Carolina, USA; ^5^Boehringer Ingelheim Pharmaceuticals, Inc., Ridgefield, Connecticut, USA; ^6^Vital Statistics LLC, Chapel Hill, North Carolina, USA

**Online supplementary material**

**Evaluation of the diagnostic algorithm**

The positive predictive value (PPV) of the diagnostic algorithm used to identify patients with IPF in this study was evaluated by manual chart review in a convenience sample of eligible patients from four participating health systems that included 12 hospitals representing a range of locations, academic and community settings, and rural and urban settings. Chart abstraction was based on a case report form containing elements that help support or rule out a diagnosis of IPF, including chest CT and lung histology reports. The presence of a diagnosis associated with an alternative form of ILD (for example, rheumatological disease) was considered to rule out IPF. The PPV was calculated as the number of cases of IPF confirmed by manual chart review divided by the number of cases identified as IPF based on the code-based inclusion criteria.

Using a broad definition, where either “pulmonary fibrosis” or “idiopathic pulmonary fibrosis [IPF]” was considered adequate for confirmation, 218 of 294 patients were confirmed to have IPF, yielding a PPV of 74.1%. The PPV was similar in male and female patients (76.0% and 72.1%, respectively). The PPV varied from 64% to 92% across hospitals. Using an alternative definition, which required use of the term “idiopathic” in the medical record to confirm the diagnosis, yielded a PPV of 57.8%.

**Case report form**


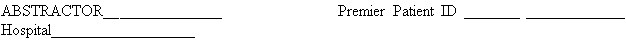


***Case Report Form***

Date of Admission* / / Today’s Date / /

*admission date must be between October 1, 2011 and October 14, 2014

| **Chart Abstraction for IPF Diagnosis Verification**  **Protocol Version 4.0 date December 4, 2017** | | | |
| --- | --- | --- | --- |
| **Diagnosis of IPF confirmed with presence of criteria 1, 2, 3 OR 1, 2 , 4 OR 1-4** | | | |
| **1.** Confirm if diagnosis of idiopathic pulmonary fibrosis (IPF) AND/OR pulmonary fibrosis is present, during the index encounter or within one year prior to the index encounter date, in any of the following (criterion 1 satisfied if **any** are marked “yes”): |  |  |  |
| 1.1. Admission note/diagnosis list | - Yes | - No | - NA |
| 1.2. Discharge summary/diagnosis list | - Yes | - No | - NA |
| 1.3. Inpatient pulmonary consult note | - Yes | - No | - NA |
| - 1. Pulmonary clinic note      1. Most Recent      2. Any | - Yes - Yes | - No - No | - NA - NA |
| 1.5. Non-pulmonary clinic note | - Yes | - No | - NA |
| - Check if ONLY “pulmonary fibrosis” is present (not specified as “idiopathic” in any reviewed medical records listed above) |  |  |  |

NA = Not Available

1. Confirm that no alternative cause of pulmonary fibrosis/interstitial lung disease is present, during the index encounter or within one year prior to the index encounter date, in inpatient/outpatient problem lists, notes, or past medical history:

**If an alternative cause IS present, check if any of the following specific diagnoses are present:**

# Autoimmune/inflammatory disorders

- Rheumatoid arthritis/rheumatoid lung
- Systemic lupus erythematosus
- Systemic sclerosis/scleroderma
- Sjogren’s disease/syndrome
- Dermatomyositis/polymyostis
- Anti-synthetase syndrome
- Mixed or Undifferentiated connective tissue disease
- Wegener’s granulomatosis (aka granulomatosis with polyangiitis)
- Sarcoidosis
- None

# Environmental/occupational lung disease

- Pneumoconiosis (e.g. coal workers’ pneumoconiosis, silicosis, hard metal

pneumonconiosis)

- Asbestosis
- Radiation fibrosis/radiation pneumonitis
- Drug toxicity-related lung disease (e.g. bleomycin, amiodarone)
- Hypersensitivity pneumonitis (aka extrinsic allergic alveolitis)
- None

# Alternative interstitial lung disease diagnosis:

- Non-specific interstitial pneumonia (NSIP)
- Cryptogenic organizing pneumonia (COP—*not COPD*)
- Acute interstitial pneumonia (AIP, aka Hamman Rich Syndrome)
- Respiratory bronchiolitis-interstitial lung disease (RB-ILD)
- Desquamative interstitial pneumonia (DIP)
- Lymphoid interstitial pneumonia (LIP)
- Pleuroparenchymal fibroelastosis (PPFE)
- None
- **Check if NO ALTERNATIVE CAUSE PRESENT**

| **3.** Confirm if the following are present in chest CT report (or spiral chest CT or high -resolution chest CT [HRCT]). Use the most recent CT report available, preferably within the index encounter. If no CT report is present from the index encounter, use the most recent CT report from up to one year prior to the index encounter date (criterion 3 met if “consistent  with/diagnostic of definite UIP OR possible UIP). | | | |
| --- | --- | --- | --- |
| **DATE OF CT REPORT REVIEWED: __/__ /____**  **TYPE OF CT REVIEWED:**   - High-resolution chest chest (HRCT) - Chest CT with intravenous (IV) contrast - CT angiogram (a.k.a. PE protocol) - Routine chest CT (not meeting any of the above   descriptions)   - Lung cancer screening chest CT - Low-Dose Computed Tomography (LDCT) | - **CHECK IF NO CT REPORT AVAILABLE** | | |
| 3.1. Diagnosis of “definite UIP (usual interstitial pneumonia)” or “diagnostis of UIP” in body or conclusion | - Yes | - No | - NA |
| 3.2. Diagnosis of “possible UIP (usual interstitial pneumonia)” or “compatible with IPF” in body or conclusion | - Yes | - No | - NA |
| Supportive features in body of report (supportive data, not required for criterion 4 to be met): |  | - No - No - No | - NA - NA - NA |
| - Subpleural, basilar predominance | - Yes |  |  |
| - Reticular abnormality/change and/or septal thickening | - Yes |  |  |
| - Honeycombing | - Yes |  |  |

| **4.** Confirm if the following are present in lung histopathology report (from surgical/ “open”/ “VATS”  /thoracoscopic biopsy ONLY) at any time during the  index encounter or in any medical record prior to the index encounter date:  **DATE OF PATHOLOGY REPORT REVIEWED:**  **__/__/____** | - **CHECK IF NO RELEVANT PATHOLOGY REPORT**   **AVAILABLE** | |
| --- | --- | --- |
| Diagnosis of “UIP (usual interstitial pneumonia)” or “consistent with UIP” or “UIP pattern” in body or conclusion (criterion 4 met if YES) | - Yes | - No |
| Supportive features in body of report (supportive data, not required for criterion 4 to be met): |  |  |
| - Honeycombing | - Yes | - No |
| - Patchy (or “variegated”) fibrosis | - Yes | - No |
| - Subpleural/paraseptal distribution | - Yes | - No |
| - Fibroblastic foci | - Yes | - No |

**OFEV drug usage**

1. Does the medical record state that the participant is taking, or has ever previously taken nintedanib (OFEV®)?
   - No
   - Yes (**if yes, please also complete the supplemental case report form on page 6**)

**Person verifying eligibility criteria:**

*Printed name Signature*

*Date*

# *Diagnosis of IPF confirmed with presence of criteria 1, 2, 3 OR 1, 2, 4 OR 1-4.

## *Supplemental Case Report Form – OFEV Use*

**INSTRUCTIONS**: Complete this supplemental page only if the answer to question 5 on page 5 (“Does the medical record state that the participant is taking, or has ever previously taken OFEV® (nintedanib)?”) of the Case Report Form is “Yes.”

| Supplemental Questions for IPF Records with Nintedanib Use | |
| --- | --- |
| 1. Confirm that the medical record states that the participant is taking, or has ever previously taken nintedanib (OFEV®) | - Yes ❑ No (If “No,” do not continue.) |
| 2. If yes to #1, what is the date of the first note (admission note, discharge summary, clinic note, consultation note) that states that the patient is taking or has previously taken nintedanib? | **DATE OF 1st Note Indicating Nintedanib Use:**  **/ /** |
| 3. If yes to #1, when did the patient start taking nintedanib? (allow day/month/year, month/year, year only, and “unknown or not stated”) | **DATE OF 1st Recorded Nintedanib Use:**  **/ /** OR ❑ Unknown  / Not Started |
| 4. If yes to #1, did the patient stop taking nintedanib? | - Yes ❑ No |
| 5. If yes to #4, when did the patient last stop taking nintedanib (allow day/month/year, month/year, year only, and "unknown or not stated”). If the patient has started and stopped multiple times, record only the last stop date. | **DATE OF Last Nintedanib Use:**  **/ /** OR ❑ Unknown  / Not Started |
| 6. Is patient death recorded in the abstracted portion of the medical record? | - Yes ❑ No |
| 7. If yes to #6, was the patient taking nintedanib at the time of death? (allow yes, no, unknown) | - Yes ❑ No ❑ Unknown |

**Person verifying Nintedanib Use / SAE:**

*Printed name Signature*

*Date*

**Supplementary Table. Identification of characteristics**

| **Characteristic** | **Identification codes/source data** |
| --- | --- |
| Mechanical ventilation |  |
| Invasive mechanical ventilation | ICD-9: 96.7, 96.71, 96.72, 31.2, 31.21, 31.29, 96.04, 96.05, V44.0; CPT/HCPCS: K0165, 31502, 31600, 31601, 31603, 31605, 31610, 31611, 31612, 31613, 31614 |
| Non-invasive mechanical ventilation | ICD-9: 93.9 |
| Unknown mechanical ventilation | ICD-9: V46.1, V46.11, V46.12, V46.13, V46.14, 997.31 |
| Chest CT; HRCT | CPT: 71250, 71260, 71270 |
| Surgical lung biopsy, other lung biopsy | CPT: 32095, 32096, 32098, 32405, 32602, 32607; ICD-9: 33.25 to 33.28; billing descriptions |
| Bronchoscopy | ICD-9: 33.27; billing descriptions |
| Echocardiogram | ICD-9: 37.28, 11.24, 88.73; billing descriptions |
| Concurrent diagnoses |  |
| Acute heart failure | ICD-9: 428.21, 428.23, 428.31, 428.33, 428.41, 428.43 |
| Cerebrovascular disease | ICD-9: 430.xx to 438.xx |
| Chronic obstructive pulmonary disease | ICD-9: 490.xx - 491.xx, 492.xx, 494.xx, 496.xx |
| Congestive heart failure | ICD-9: 428.xx |
| Coronary artery disease | ICD-9: 414.01, 414.0x |
| Diabetes | ICD-9: 250.xx |
| Diverticulosis | ICD-9: 562.xx |
| Gastroesophageal reflux disease | ICD-9: 530.81x |
| Hyperlipidaemia | ICD-9: 272.4x, 272.2xx |
| Hypertension | ICD-9: 401.xx |
| Lung cancer | ICD-9: 162.xx |
| Malnutrition | ICD-9: 262, 263, 263.0, 263.1, 263.8, 263.9x |
| Pneumonia | ICD-9: 481, 482, 482.1, 482.2, 482.3, 482.31, 482.32, 482.39, 482.4, 482.41, 482.42, 482.49, 482.81, 482.82, 482.83, 482.84, 482.89, 482.9, 483.1, 483.8, 484.8, 485, 486, 487.0, 487.1, 487.8, 488.0, 488.1 |
| Pneumothorax | ICD-9: 512.xx |
| Renal failure | ICD-9: 584.xx |

CPT, Current Procedural Terminology; HCPCS, Healthcare Common Procedure Coding System; ICD-9, International Classification of Diseases Ninth Revision.
